# Phosphatidylinositol 3-Kinase Plays a Vital Role in Regulation of Rice Seed Vigor via Altering NADPH Oxidase Activity

**DOI:** 10.1371/journal.pone.0033817

**Published:** 2012-03-20

**Authors:** Jian Liu, Jun Zhou, Da Xing

**Affiliations:** MOE Key Laboratory of Laser Life Science and Institute of Laser Life Science, College of Biophotonics, South China Normal University, Guangzhou, China; Fundação Oswaldo Cruz, Brazil

## Abstract

Phosphatidylinositol 3-kinase (PI3K) has been reported to be important in normal plant growth and stress responses. In this study, it was verified that PI3K played a vital role in rice seed germination through regulating NADPH oxidase activity. Suppression of PI3K activity by inhibitors wortmannin or LY294002 could abate the reactive oxygen species (ROS) formation, which resulted in disturbance to the seed germination. And then, the signal cascades that PI3K promoted the ROS liberation was also evaluated. Diphenylene iodonium (DPI), an NADPH oxidase inhibitor, suppressed most of ROS generation in rice seed germination, which suggested that NADPH oxidase was the main source of ROS in this process. Pharmacological experiment and RT-PCR demonstrated that PI3K promoted the expression of Os *rboh*9. Moreover, functional analysis by native PAGE and the measurement of the 2, 3-bis-(2-methoxy-4-nitro-5-sulfophenyl)-2H-tetrazo-lium-5- carboxanilide (XTT) formazan concentration both showed that PI3K promoted the activity of NADPH oxidase. Furthermore, the western blot analysis of OsRac-1 demonstrated that the translocation of Rac-1 from cytoplasm to plasma membrane, which was known as a key factor in the assembly of NADPH oxidase, was suppressed by treatment with PI3K inhibitors, resulting in the decreased activity of NADPH oxidase. Taken together, these data favored the novel conclusion that PI3K regulated NADPH oxidase activity through modulating the recruitment of Rac-1 to plasma membrane and accelerated the process of rice seed germination.

## Introduction

Seed germination is a complex event, which commence with the uptake of water by the quiescent dry seed and terminate with the elongation of the embryonic axis [1]. This process is influenced by various factors, including some signaling molecules, such as reactive oxygen species [2] and NO [3], several plant hormones, such as abscisic acid (ABA) [4], gibberellic acid (GA) [5] and ethylene [6], and other factors, for instance, HCN [7] and water channel proteins [8]. In addition, some lipids, for example, Phospholipase D (PLD) and its product phosphatidic acid (PA), are also indicated to be involved in seed germination [9]. PI3K as a key role in the regulation of lipid signal has been reported in plant physiology. However, there is no thorough study on the relationship between PI3K and seed germination.

In mammalian cells, there are three types of PI3K with different substrate specificities [10]–[12]. In plant cells, only type III PI3K, which phosphorylates the D-3 position of phosphoinositides, has been identified. The product of PI3K is Phosphatidylinositol 3-phosphate (PI3P), which is present at very low level in plant cell but turn over rapidly following the alteration of external environment [13]–[17]. PI3K and its product have been proved to be involved in various physiological events and stress responses [18], including root hair growth [19], root nodule formation [20], stomatal closing movement [21], nuclear transcription [12], auxin-induced reactive oxygen species production and root gravitropism [22], pollen development [23], actin filament reorganization [24], endocytosis of plasma membrane and the salt-stress-induced production of ROS [25]. However, in seed germination, there is no elaborate study about the function of PI3K. In particular, how PI3K and its downstream pathway involved in modulating seed germination remains unknown.

NADPH oxidase, which plays a significant role in generating ROS, has been implicated in seed germination [6], [8], [26]. In mammalian cells, PI3K can mediate the recruitment of the Rac-1 GTPase and the oxidase submits p47^phox^ to the cell membrane, which affects the assembly of NADPH oxidase complex and eventually the activity of NADPH oxidase [27], [28]. In addition, phosphoinositide products of PI3Ks can directly bind to the PX domains of the oxidase subunits p40^phox^ and p47^phox^, which influences their abilities to translocate p67^phox^ and finally alters the activity of NADPH oxidase [29], [30]. In plant cells, one type of PI3K related to yeast Vps34 has been identified and seems to have more extensive functions [12]. Moreover, recent findings have proved that Rho GTPase, especially Rac1, is involved in the regulation of NADPH oxidase activity in rice [31], [32]. However, there is still rare information about the association of PI3K with NADPH oxidase activity in plants.

In this study, we revealed that PI3K altered the quantity of ROS and played an essential role in rice seed germination, through the regulation of the expression and activity of NADPH oxidase. It was also characterized how PI3K regulated the activity of NADPH oxidase. Finally, we proposed a novel hypothetical model that PI3K regulated the translocation of Rac-1 from cytoplasm to plasma membrane, resulting in activated assembly of NADPH oxidase in rice seed germination.

## Results

### Changes of PI3K expression in rice seed after imbibition and the expression of PI3K is regulated by Ca^2+^

**Figure 1:**
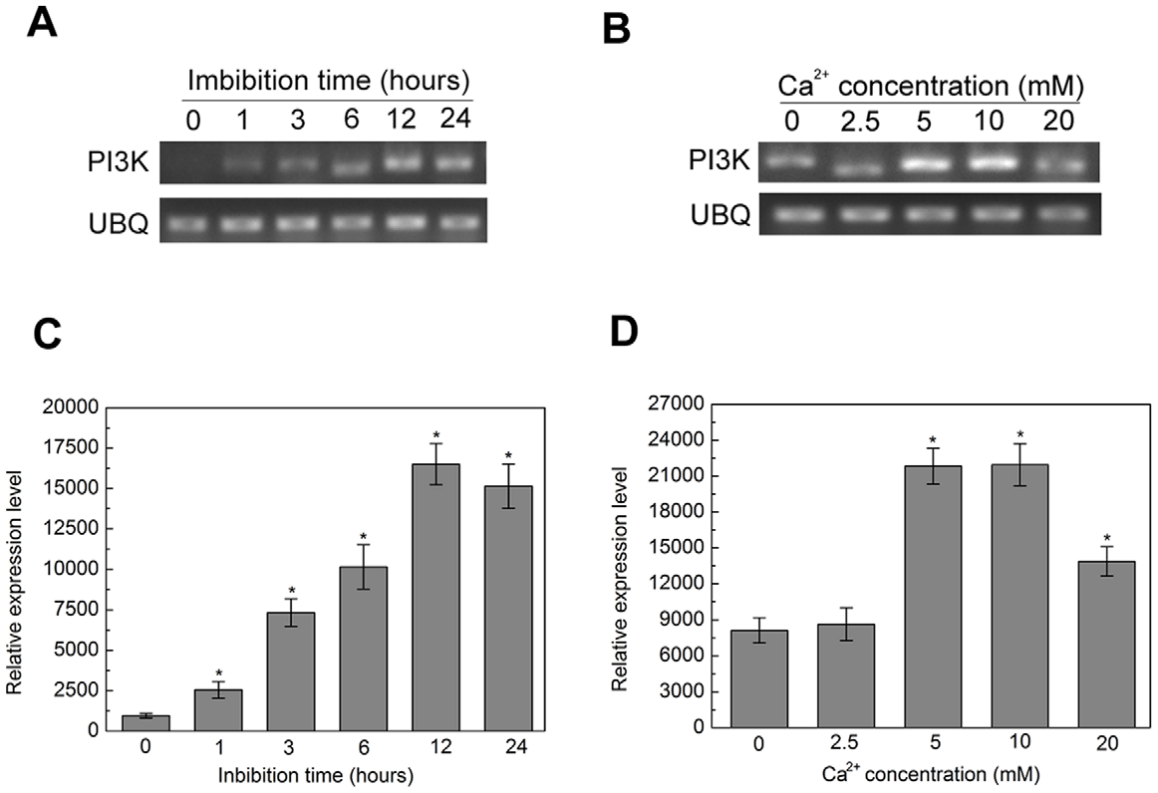
Changes of *PI3K* expression in rice seed after imbibition. RT-PCR assayed the expression of *PI3K* from rice seed, which was imbibed from 0 hour to 12 hours in the presence (A) or absence (C) of 20 mM CaCl_2_. *UBQ*, mRNA of rice ubiquitin was used as control. (C) Different concentration of CaCl_2_ was used to treat with seed in 12 hours imbibition, then isolated the embryo and analyzed the alteration of *PI3K* expression by RT-PCR. (B, D) Quantitive analysis of the result of RT-PCR in statistical method. Data are means of three replicates ± SD. * indicates the values that are significantly different from control (P<0.05).

To determine the dynamic expression of *PI3K* in rice seed germination, the total RNA was extracted from the rice seed embryo after imbibition. The result of RT-PCR analysis suggested that PI3K might involve in the regulation of seed germination. Meanwhile, it was found that the expression of *PI3K* was increased with the prolongation of imbibition time (Figure 1A). This expression peaked at 12 h and reached approximately 30 times as many as seeds without treatment (Figure 1C). Interestingly, low concentration of Ca^2+^ (below 10 mM) could further increase the expression of *PI3K* in rice seeds after germinated for 12 h. However, when the concentration of Ca^2+^ was up to 20 mM, the *PI3K* expression began to decrease slightly compared with the treatment with 10 mM CaCl_2_ (Figure 1B, D).

### LY294002 and Wortmannin inhibit rice seed germination

**Figure 2:**
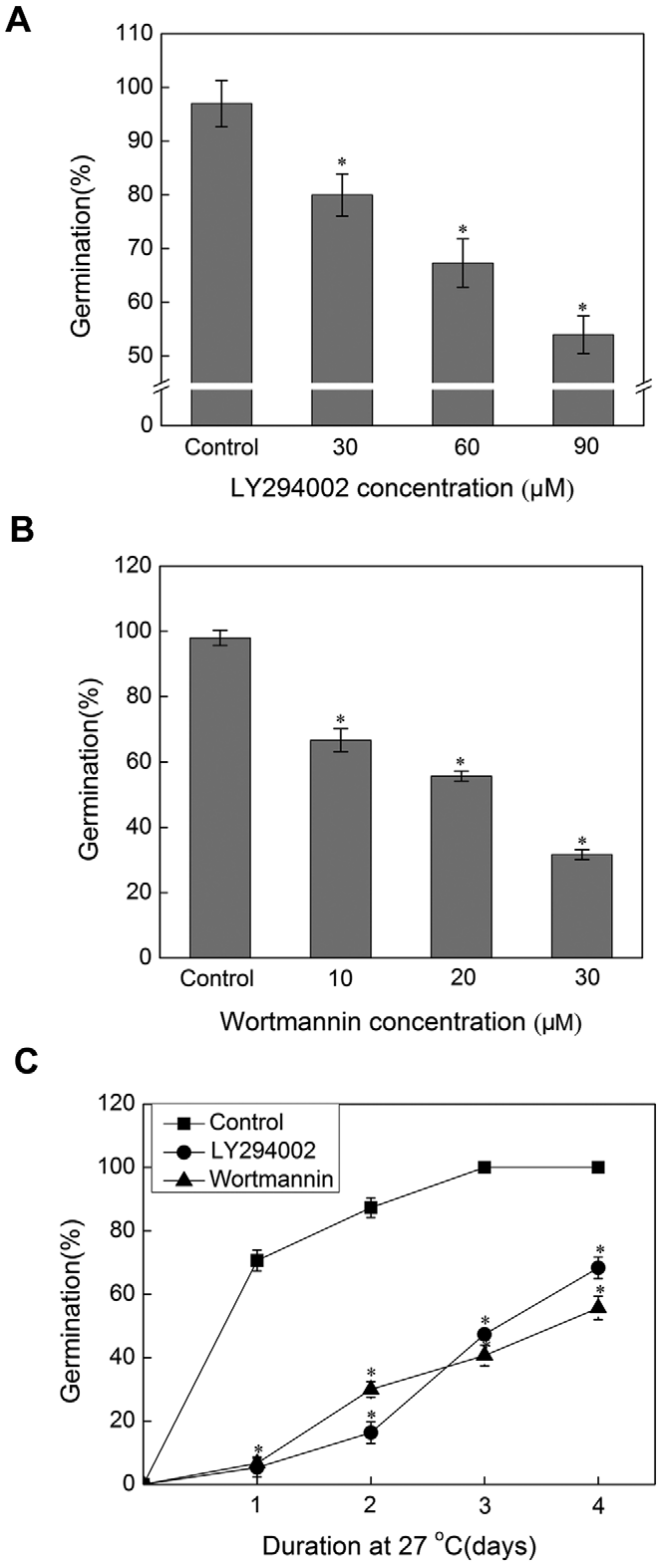
The effect of PI3K inhibitors LY294002 and Wortmannin on seed germination in rice. Germination of rice seed came from the same seed lot at 28°C in the darkness. (A) and (B) Dose-dependent effects of PI3K inhibitor LY294002 (30 µM, 60 µM, 90 µM) or Wortmannin (10 µM, 20 µM, 30 µM) on seed germination. The germination of rice seed after 5 days imbibition was counted. (C) Rice seeds treated with water containing 60 µM LY294002 or 20 µM Wortmannin. The germination of rice seed was counted during five days after imbibition. Means of three replicates ± SD. * indicates the values that are significantly different from control (P<0.05).

Next, Wortmannin and LY294002, two kinds of PI3K inhibitors with different action mechanisms, were used to test the role of PI3K in the de-coated (without pericarp) rice seed germination [21]. Following treatment with 60 µM LY294002 at 27°C for four days, the seed germination rate decreased to about 67.3% compared with the control. And it was further decreased to 54.0% when the concentration of LY294002 was increased to 90 µM (Figure 2A). The seed germination rate was 55.7% of the control in the presence of 20 µM Wortmannin and it would be decreased to 31.7% of the control with 30 µM Wortmannin (Figure 2B). Dynamics of seed germination revealed that, relative to the control, 60 µM LY294002 or 20 µM Wortmannin displayed both significant inhibitory effects on the seed germination at any time point (Figure 2C). From these results, we reasonably concluded that PI3K played a positive role in rice seed germination.

### LY294002 and Wortmannin inhibit ROS production

PI3K inhibitors suppressed the ROS production in guard cell, root hair, and pollen tube [18], [19], [23]. However, it was uncertain that PI3K inhibitors restrained the ROS formation in rice seed germination. In our case, it was found that exogenous hydrogen peroxide (H_2_O_2_) could partly rescue the inhibitory effects of PI3K inhibitors on rice seed germination (Figure S1A). This finding allowed us to assume the possible association of PI3K with ROS production during rice seed germination.

**Figure 3:**
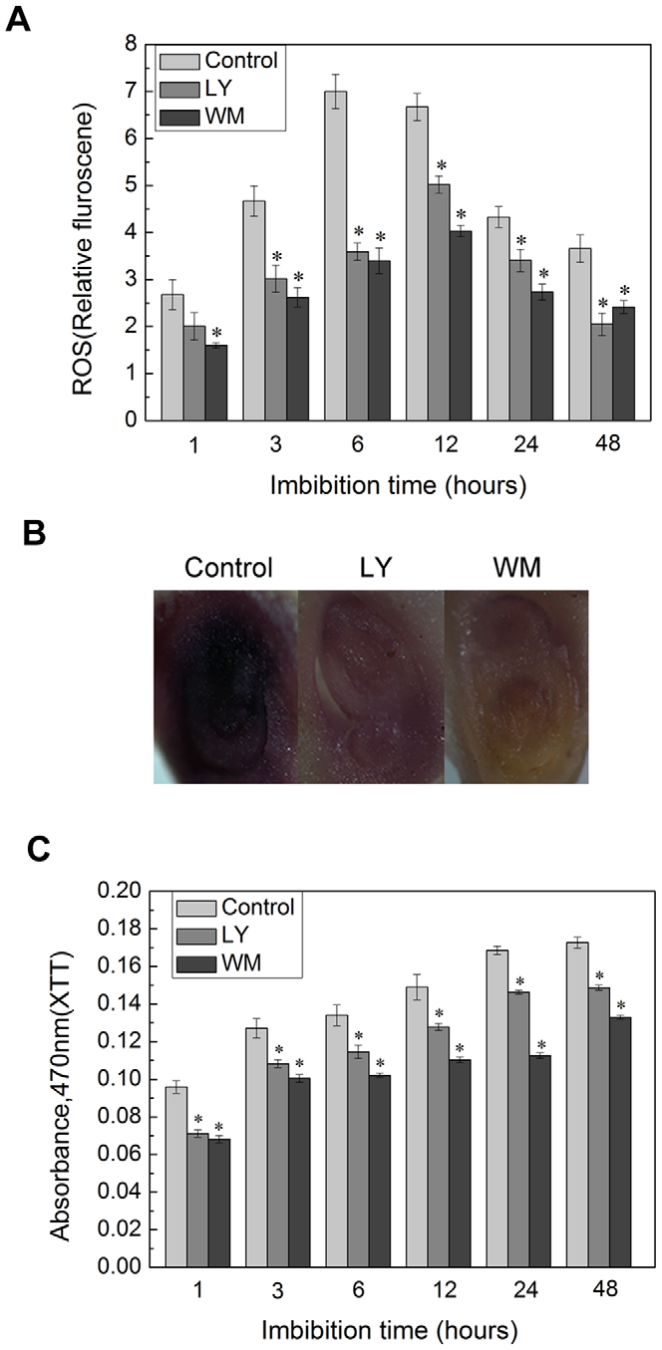
PI3K inhibitors LY294002 and Wortmannin suppressed the ROS generation in rice seed embryos during germination. Rice seed embryos was pretreated with 20 µM Wortmannin or 60 µM LY294002. (A) ROS was determinated by H_2_DCFDA, and superoxide anion (C) was examined by XTT reduction. DCF fluorescence and the production of XTT reduction absorbance were measured using a microtiter plate reader as described in Materials and Methods. (B) NBT staining analysis. Seed was imbibed for 12 hours with or without 60 µM LY294002 or 20 µM wortmannin, and then the isolated embryo was incubated with 6 mM NBT in 10 mM Tris-HCl buffer (pH 7.4) at 30°C for 2 h. Superoxide anion was visualized as deposits of dark-blue insoluble formazan compounds. Microscopic images were taken using a Zeiss microscope as described previously. Pictures represent typical examples. Means of three replicates ± SD. * indicates the values that are significantly different from control (P<0.05).

To further investigate this relationship between PI3K and ROS, ROS probe H_2_DCFDA was used to examine the characteristics of ROS production under the treatment of pharmacological inhibitors of PI3K LY294002 (60 µM) or Wortmannin (20 µM). Results demonstrated that, during the imbibition period of 48 h, treatment with PI3K inhibitors resulted in lower level of ROS production compared with the control (Figure 3A). Meanwhile, the change of superoxide anion was also examined through NBT staining [33] and XTT test [34]. As shown in Figure 3B, 3C, the alteration of superoxide anion was similar with the change of ROS level. Interestingly, PI3K inhibitors seemed to suppress ROS not only by NADPH oxidase but also other sources (Figure S2). The above findings led us to get the conclusion that PI3K inhibitors could suppress the formation of ROS in rice seed germination.

### NADPH oxidase is an important source of ROS in rice seed germination

Several studies have showed that ROS plays a key role in seed germination [7], [35], [36]. Here, we also found that rice seed germination could be inhibited by ROS scavengers, 10 mM KI or 1 Mm ASA (Figure S1B). Subsequently, 100 µM diphenylene iodonium (DPI), one of the highly effective inhibitor of NADPH oxidase [27], was used to explore whether NADPH oxidase is involved in ROS production responsible for rice germination. As shown in Figure S1B, the germination rate in rice seed treated with DPI was lower than that of control. Interestingly, exogenous H_2_O_2_ (20 mM) could partly rescue the decrease in seed germination caused not only by KI and ASA but also by DPI (Figure S1B). These phenomenons indicated that NADPH oxidase might be the important source of ROS formation in rice seed embryo under germination.

**Figure 4:**
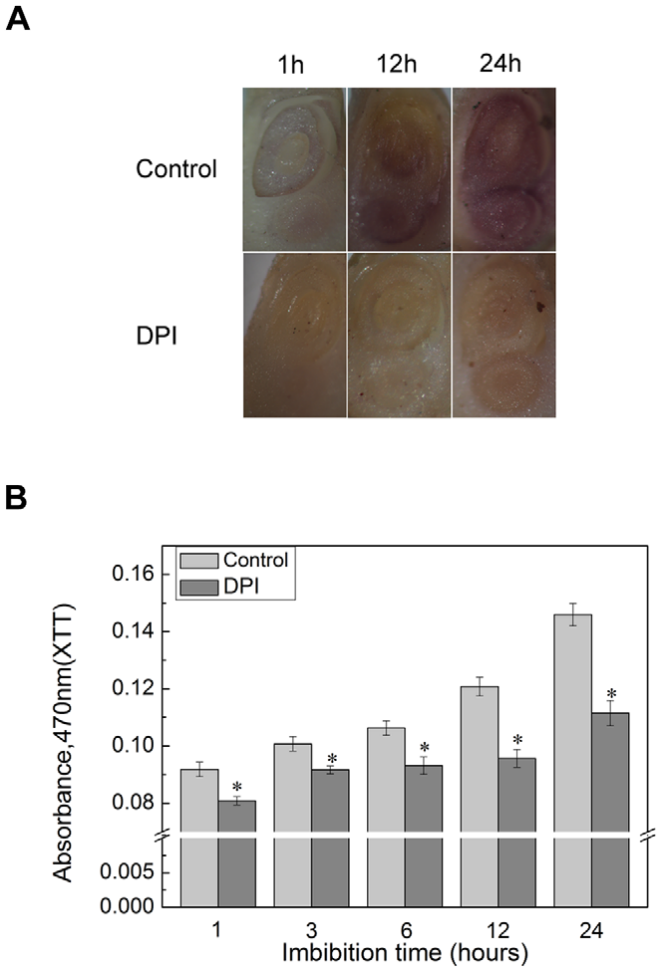
NADPH oxidase is the important source of ROS in seed germination. (A) Rice seed imbibed for several hours in the presence or absence of 100 µM DPI and then isolated the seed embryo, NBT staining analysis was performed according to the description in materials and methods. (B) XTT was used to assay the superoxide anion with or without 100 µM DPI. Pictures represent typical examples. Values are means of three replicates ± SD. * indicates the values that are significantly different from control (P<0.05).

To prove this hypothesis, the concentration of superoxide anion was under determination in the absence and presence of 60 µm LY294002 or 20 µm Wortmannin. As shown in Figure 4A, NBT was applied to evaluate the quantity of superoxide anion. DPI (100 µM) was added before NBT staining. Compared with control, exogenous DPI could significantly suppress the formation deposits in the embryos.

To further verify this phenomenon, superoxide anion was quantified by XTT [34]. As was illustrated in Figure 4B, under the treatment of PI3K inhibitors LY294002 (60 µM) or Wortmannin (20 µM), the formation of superoxide anion was obviously restricted in contrast with control. These observations confirm the idea that NADPH oxidase is an important source of ROS in seed germination.

### LY294002 and Wortmannin inhibit the expression of NADPH oxidase

**Figure 5:**
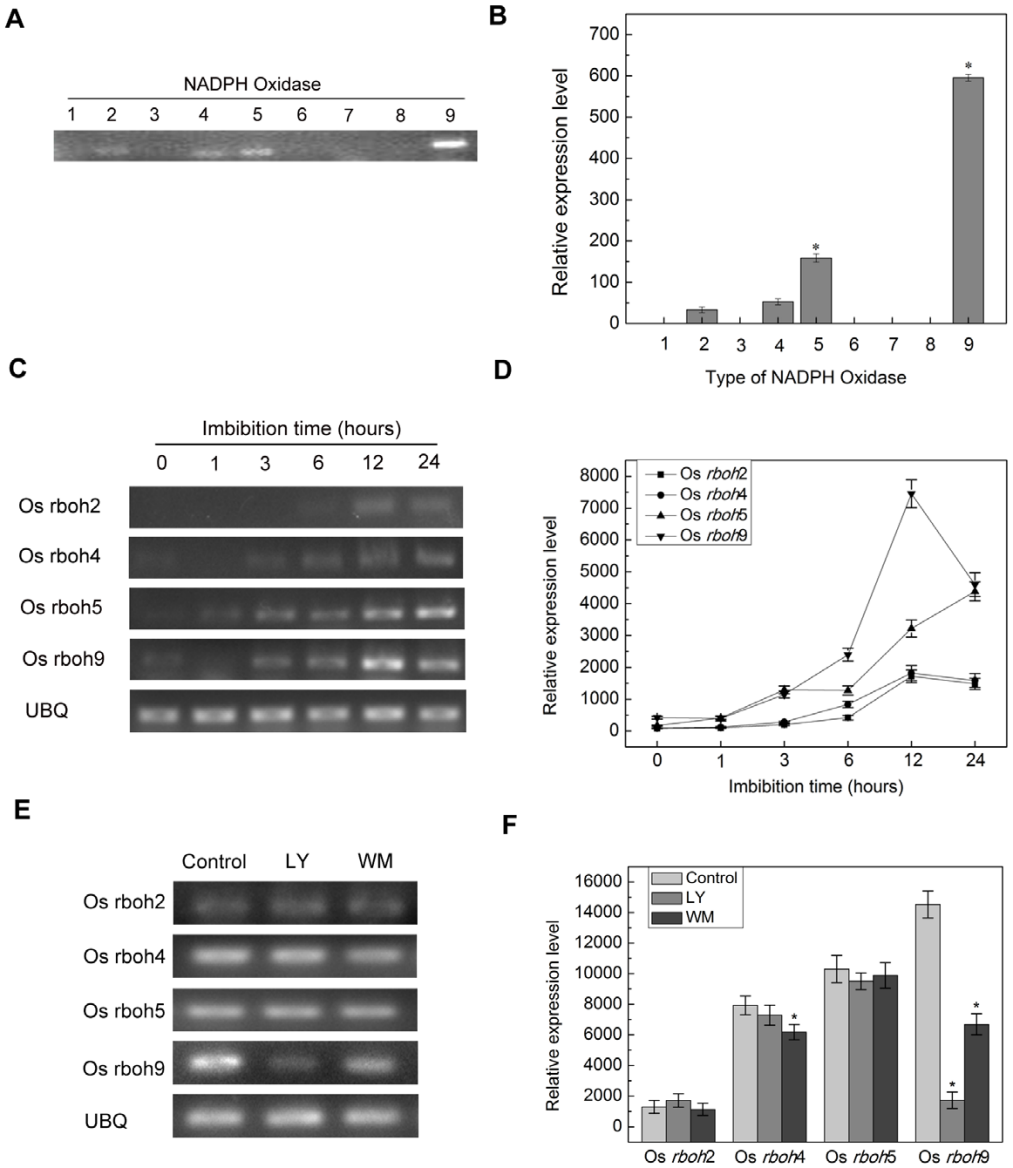
PI3K inhibitors abate the expression of NADPH oxidase in rice seed embryos during germination. (A). The expressive type of Os *rboh* gene from rice seed. RT-PCR was performed with 30 cycles for all Os *rboh*, *UBQ* was used as control (C) The expression of Os *rboh*2, Os *rboh*4, Os *rboh*5 and Os *rboh*9 were examined following the prolongation of imbibition time (E) Seed was incubated with 60 µM LY294002 or 20 µM wortmannin, then isolated the seed embryo and extracted the total RNA, The expression of *rboh*2, *rboh*4, *rboh*5, *rboh*9 were determined. (B, D, F) Quantitive analysis of these results of RT-PCR in statistical method. Pictures represent typical examples and the data are mean ± SD of three repeats. * indicates the values that are significantly different from control (P<0.05).

The earlier studies have reported that NADPH oxidase-generated ROS plays a key role in seed germination [7], [35], [36]. As mentioned above, PI3K inhibitors can prevent ROS production during rice seed germination. Therefore, it is worth assuming that PI3K might control ROS level via regulating NADPH oxidase. In the previous study, it has been proved that PI3K is associated with nuclear transcription sites in higher plant [12]. Thus, we examined whether PI3K regulated the transcription of NADPH oxidase in rice seed germination. Firstly, in order to investigate the types of NADPH oxidase in the rice seed germination, the RNA was extracted from wild type rice seed imbibed for 24 h. RT-PCR experiments revealed that NADPH oxidase Os *rboh*2, Os *rboh*4, Os *rboh*5 and Os *rboh*9 played a primary role in rice seed germination (Figure 5A, B). Besides, the dynamics of these types of NADPH oxidase expression was also assayed during the initial 24 h of rice seed germination (Figure 5C, D). To investigate whether PI3K promote the transcription of NADPH oxidase in the rice seed germination, the expression of NADPH oxidase was examined in the presence of 60 µm LY294002 or 20 µm Wortmannin. As shown in Figure 5E, F, the PI3K inhibitors notably restrained the expression of NADPH oxidae Os *rboh*9 and slightly suppressed the expression of Os *rboh4* in comparison with other types of NADPH oxidase. On the basis of the above results, we drew the conclusion that PI3K could promote the transcription of Os *rboh4* and Os *rboh*9 other than the rest types of NADPH oxidase.

### PI3K inhibitors suppress the activity of NADPH oxidase

**Figure 6:**
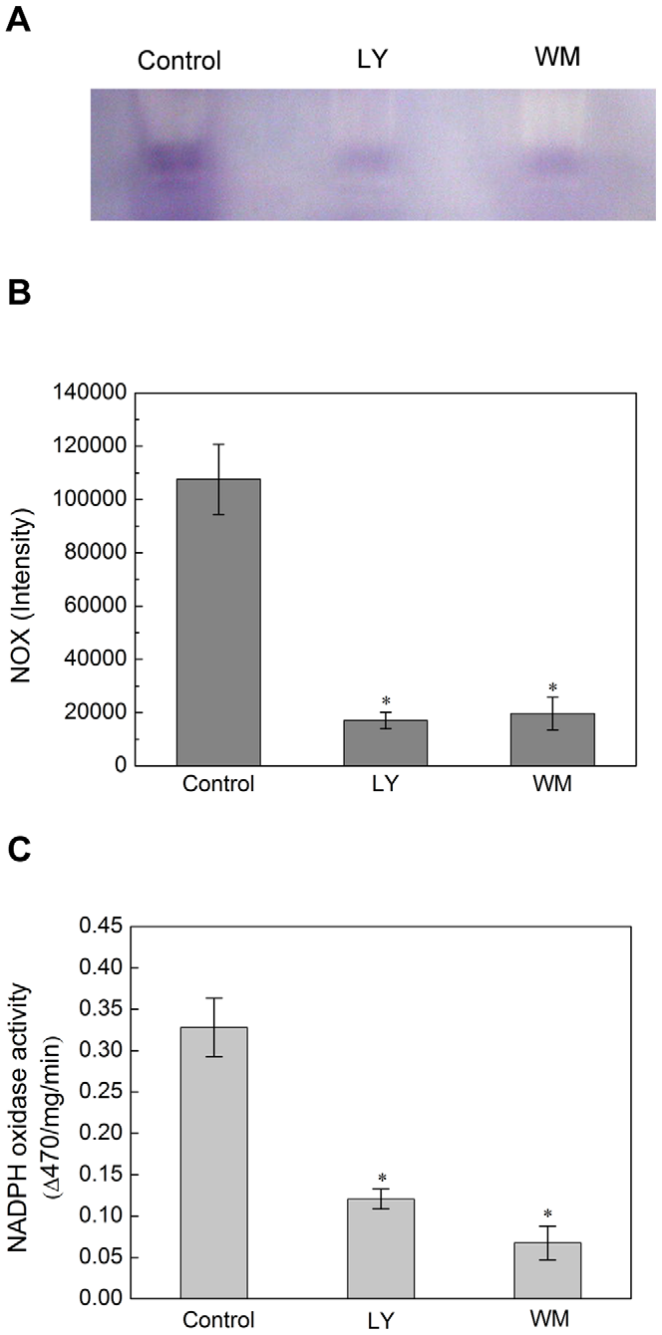
PI3K inhibitors suppress the activity of NADPH oxidase in seed germination. (A) In situ gel NBT assay. The PM fraction was separated by native PAGE, and the gel was incubated with NBT solution, then with 0.2 mM NADPH until the appearance of blue formazan bands was observed. The reaction was stopped by immersion of the gel in distilled water. (B) Quantitive analysis of the result of native PAGE in statistical method. (C) The seed embryos was immersed in the solution within the PI3K inhibitor for 12 hours, then isolate the plasma membrane, Plasma membrane vesicles (4 mg protein) were incubated with XTT at 25°C for 10 min. The reaction solution was used for spectrophotometric analysis of XTT formazan absorbance at A470. NADPH oxidase activity was expressed as ΔA470 per mg protein per min (ΔA470 represents the difference of XTT formazan absorbance at 470 nm in the presence and absence of superoxide dismutase [SOD]). Means of three replicates ± SD and the pictures represent typical examples. * indicates the values that are significantly different from control (P<0.05).

The effect of PI3K on the activity of NADPH oxidase was also examined. At first, *in situ* gel NBT was performed with the isolated plasma membrane, which was obtained from the rice seed embryo imbibed for 24 h in the absence and presence of 60 µM LY294002 or 20 µM Wortmannin. As shown in Figure 6A, the band from the control PM was obviously stronger than that from the PM in the presence of 60 µM LY294002 or 20 µM Wortmannin. And then, the NADPH oxidase enzymatic activity was also examined by the measurement of the XTT formazan concentration with 60 µM LY294002 or 20 µM Wortmannin. The experimental result showed that the LY294002 and Wortmannin inhibited NADPH oxidase activity by 36.6% and 18.2%, respectively (Figure 6B). It appears therefore, that PI3K promoted the activity of NADPH oxidase in rice seed germination.

### PI3K is required for subcellular translacaiton of Rac-1

In mammalian cells, it has been proved that PI3K class I can regulate the translocation of cytosolic factors, such as Rac-1. In fact, it is necessary for the assembly of the active NADPH oxidase complex to translocate Rac-1 to the cell membrane in rice cells [31]. However, it has been unclear whether PI3K regulates NADPH oxidase activity through mediating the translocation of Rac-1.

**Figure 7:**
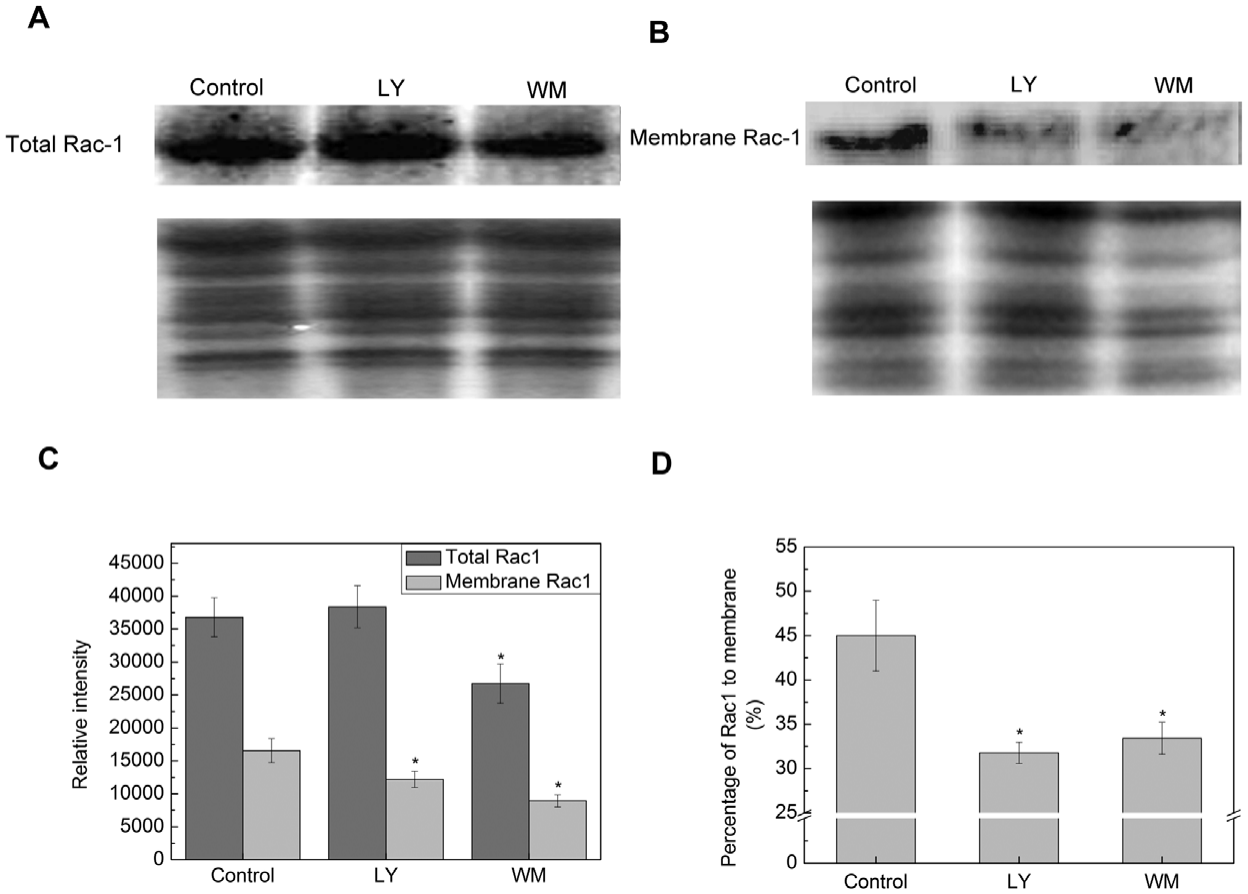
PI3K inhibitors inhibit the translocation of Rac-1 to the Plasma membrane. (A) Total protein and (B) membrane protein were respectively extracted as description in materials and methods. The proteins were separated by SDS-PAGE and subjected to Western blot analysis using antisera which recognize Rac-1. (C) Densitometric analysis of the western blots as shown in (A) and (B). (D) The percentage of Rac-1 which was transmitted to plasma membrane. Data represent means ± SD from three independent experiments. * indicates the values that are significantly different from control (P<0.05).

To investigate the possible mechanisms of Rac1 regulated by PI3K, western blot was used. The experiment was performed with total or membrane protein isolated from rice seed embryo cells imbibed for 24 h with or without 60 µM LY294002 or 20 µM Wortmannin. Western blot analysis of membrane protein indicated that the translocation of Rac-1, following the treatment of LY294002, was suppressed compared with control, whereas the quantity of Rac-1 from the total protein was not obviously altered. In addition, compared with that of LY294002, another PI3K inhibitor Wortmannin seemed to abate the amount of total Rac1 while it reduced the amount of membrane Rac1 severely (Figure 7A, 7B). These results (Figure 7C, 7D) confirmed the speculation that PI3K promoted the translocation of Rac-1 to the membrane and thus facilitated the activity of NADPH oxidase.

## Discussion

Although many papers reported that the seed of PI3K mutant showed a reduced germination rate compared with wild type [23], it has rarely been directly assayed so far. In this study, we elaborately characterized the effects of PI3K on the rice seed germination by promoting NADPH oxidase activity. LY294002 and Wortmannin, two sorts of PI3K specific inhibitor, obviously inhibited the rice seed germination. Our mainly study here focused on the inhibition of ROS generation (Figure 3) and the relationship between PI3K and NADPH oxidase in seed germination (Figure 6, 7).

### PI3K activity is closely correlated with rice seed germination

In plant cells, the level of PI3P, as the product of PI3K, were relatively low, but turn over rapidly following the alteration of external environment [14]. This character determined that the level of PI3P maybe has more sensitive response to the external stimulation. Thus, we firstly examined the expression of *PI3K* in rice seed germination (Figure 1A). From 0 hour to 12 hours, the expression of *PI3K* almost raised thirty times than initial expression, which obviously indicated the key role of PI3K in rice seed germination. Interestingly, the exogenous Ca^2+^ promoted the expression of *PI3K* (Figure 1B). As we know, the level of Ca^2+^ rapidly increased following by the uptake of water in the early germination. Here, PI3K seemed to be one of the important relay station of Ca^2+^ signal. In previous publication, it had been reported that Wortmannin and LY294002 inhibited Ca^2+^ oscillation induced by ABA [37], which supported the role of PI3P in ABA-induced Ca^2+^ oscillation. In contrast with the above studies, our results showed that Ca^2+^ promoted the expression of PI3K. Meanwhile, it was reported that PI3K could also raise the levels of intracellular Ca^2+^ [37]. Therefore, we speculated that PI3K and Ca^2+^ seemed to have a feedback regulated relationship. This cycle rapidly altered the total quantities of PI3K and Ca^2+^, which laid the foundation for fulfilling their functions in the seed germination.

To provide direct evidence for the role of PI3K on the rice seed germination, Wortmannin and LY294002, two kinds of PI3K inhibitors with different action mechanisms, were used to treat with rice seed (Figure 2A, B). 60 µM LY294002 inhibited the seed germination by 67.3%, while 20 µM Wortmannin suppressed the seed germination by 55.6%. In addition, this effect of PI3K inhibitors on rice seed germination was concentration dependent. To investigate the dynamic alteration of rice seed germination following by the prolongation of imbibition under the treatment with PI3K inhibitors, the germination rate from 1 day to 5 days showed that PI3K inhibitors suppressed the rice seed germination during these processes.

### PI3K-Rbohs interaction suggested a novel regulatory mechanism for rice seed NADPH oxidase

Many articles have reported that ROS played an important role in seed germination. In these processes, ROS could interplay with the hormonal signaling pathway, such as abscisic acid (ABA), gibberellins (GAs) and ethylene [35], [38]. The accumulation of ROS could be beneficial for seed germination through controlling the cell redox status. Various transcription factors had been shown to sense ROS via the formation of disulfides involving thioredoxin and glutaredoxin [7], [39]. In addition, the established mechanisms of ROS transduction pathway, which involved MAP kinase cascade activation, inhibition of phosphatases, activation of Ca^2+^ channels and Ca^2+^ binding proteins, also had an important function in seed germination.

In previous studies, it had been reported that PI3K could regulate the formation of ROS in majority of plant tissues, such as root hair [19], pollen tube [23] and guard cell [21]. Our experiments proved that the exogenous H_2_O_2_ abated the inhibition of PI3K inhibitors during the rice seed germination (Figure S1A), which implied that ROS played a significant role by the regulation of PI3K. Thus we investigated whether PI3K promoted the generation of ROS in rice seed germination. H_2_DCFDA was used to determine the quantity of ROS. In contrast with the treatment of control, the ROS quantity of treatment was notably decreased. It seemed that PI3K inhibitors inhibited ROS formation. To further verify this point, superoxide anion was examined by NBT dyeing and the determination of XTT reduction product absorbance. Similar observation was obtained. Meanwhile, NADPH oxidase was also proved to be the important source of ROS in rice seed germination when used DPI, an NADPH oxidase inhibitor. On the basis of these results, the clearly signal pathways between PI3K and NADPH oxidase was put on the agenda.

In the former publication, it had been shown that PI3K activity was associated with nuclear transcription [12]. Thus, we firstly investigated whether PI3K regulated the transcription of NADPH oxidase. From the analysis of the rice genome database, nine Os *rboh* genes were identified. RT-PCR analysis showed that only *rboh*2, *rboh*4, *rhoh*5 and *rboh*9 were expressed in rice seed germination. The determination of the NADPH oxidase expression under the treatment with PI3K inhibitors verified that PI3K inhibitors inhibited the expression of *rboh4* and *rboh*9. It seemed that PI3K could affect the formation of ROS by regulating *rboh4* and *rboh*9 expression.

At the same time, the influence of PI3K inhibitors on the activity of NADPH oxidase was also performed. Two approaches, native PAGE and the determination of XTT reduction product absorbance, both demonstrated that PI3K inhibitors obviously suppressed the activity of NADPH oxidase.

However, how PI3K regulates the activity of NADPH oxidaes is still unknown. In human myeloid cell, PI3P was associated with the noncatalytic component p40^phox^ of the NADPH oxidase, and then affected the translocation of p40^phox^ to p67^phox^, finally altered the activity of NADPH oxidase [29]. Moreover, in phagocytic cell, PI3K could influence that plasma membrane recruited Rac-1 and p40^phox^ [27], which were the necessary components of NADPH oxidase. The homolog of p40^phox^ has not been reported in plant, but Rac-1 had been proved as an important role on the activity of NADPH oxidase in rice. Therefore, it was necessary to investigate on the relationship between PI3K and Rac-1.

The analysis of western-blot was shown by using Rac-1 antibody. As expected, under control conditions, the percentage of Rac-1, which was transmitted to membrane, was more than that of inhibitor treatment. The translocation of Rac-1 to the membrane was suppressed, but total Rac-1 in the homogenate was not changed obviously under the treatment of LY294002. In addition, compared with that of LY294002, another PI3K inhibitor Wortmannin seemed to alter the amount of total Rac1 while it reduced the amount of membrane Rac1 severely (Figure 7). The difference in the drug used might be result in the difference. As we know, LY294002 is a special inhibitor of PI3K, but Wortmannin is a fungal toxin that inhibits not only PI3K but also PI4K and PIPK [19]. Based on these results, it was proved that PI3K promoted the activity of NADPH oxidase through mediating the translocation of Rac-1 to membrane. However, it would lead to a new question how PI3K regulate the translocation of Rac-1. In neutrophils, P-Rex, the member of Rho family GEFs, was strongly and directly activated by PtdIns(3,4,5)P_3_. And P-Rex also was the PI3K effectors, which led to the accumulation of activated Rac (Rac-GTP) [40]. Further study will be required to identify whether or not Rac activity is influenced by PtdIns3P in plant cells, similarly as Rac GTPase was regulated by PtdIns(3,4,5)P_3_ in animal cells. Moreover, in the early seed germination, a temporary structural perturbation, particularly to membranes, was occurred following the influx of water into the cells of dry seeds [1]. As we know, PI3K participated in different stages of vesicular trafficking which play a crucial role in the process of repair. Therefore, the addition mechanisms that PI3K regulates the seed germination through vesicular trafficking remain to be investigated.

**Figure 8:**
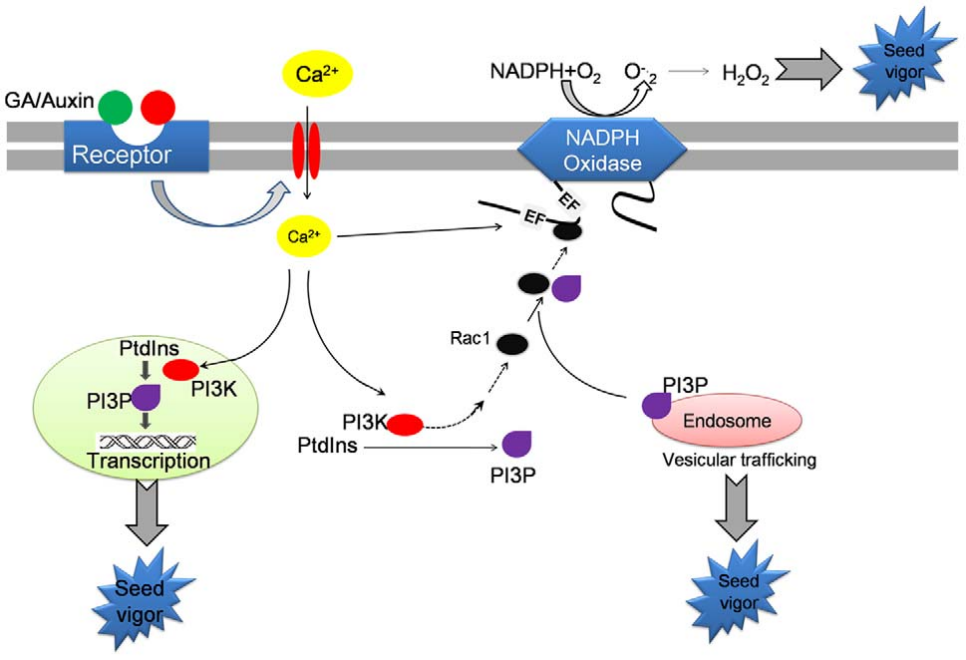
Working model of PI3K activation of Rboh in rice seed embryo. Imbibition activates the phosphoinositide signaling pathway through inducing of the PI3K activity, which plays a central role in the activation of NADPH oxidase and generation of ROS that act in the germination of rice seed.

Together with the above work, the data presented here provided a comprehensive view of the mechanism of PI3K-dependent seed germination with ROS as key signaling elements in rice seed germination. A scheme showing this mechanism was presented in Figure 8. We proposed that PI3K could promote the translocation of Rac-1 to membrane, and then elevated the activity of NADPH oxidase, finally increased the formation of ROS. The elevated ROS level could trigger carbonylation of proteins and this was specifically associated with seed germination [6].

## Materials and Methods

### Plant material and chemicals

Rice (*Oryza sativa* L. Nipponbare) seeds were kindly provided by Professor L.X. Zeng (Guangdong Academy of Agricultural Sciences, China). H_2_DCFDA was obtained from Molecular Probes (Eugene, OR, USA). DPI, nitroblue tetrazolium (NBT) and XTT were purchased from Sigma-Aldrich (Shanghai, China). LY294002 and Wortmannin were obtained from BiYunTian (Nantong, China). Rac-1 antibody was bought from Cell Signalling Technology (USA).

### Germination Tests

Dehulled rice seeds were sterilized in 70% ethanol for 5 min and then in 30% sodium hydrochlorite for 30 min. Seeds were rinsed several times with sterilized water [41]. Germination tests were performed with dehulled seeds on the moist filter paper in 9 cm Petri dishes (100 seeds per dish), which were wrapped with parafilm during the experiment, at 28°C in darkness. Seeds were considered as germinated when their radicles pierced the seed coat by 3 mm [7], [8].

To test the effect of PI3K inhibitors on seed germination, LY294002 and Wortmannin at different concentration were used to incubate rice seeds at 28°C. In order to verify the importance of ROS on seed germination, two ROS scavenger ascorbic acid (ASA) (100 mM) and KI (10 mM) were used to treat the seeds. In addition, DPI (100 µM) was also used to test if NADPH oxidase was one of the major ROS sources. The average germination rate of the triplicate of 100 seeds was calculated every 24 h over a period of 5 days.

### Total RNA isolation and RT-PCR

Embryos after being isolated from dry seeds (kept in 4°C) and germinating seeds (incubated at 28°C for 1, 3, 6, 12 and 24 h) were frozen in lipid nitrogen and stored at −80°C until used. For each extract, 100 embryos were grounds to a fine powder in lipid nitrogen, and total RNA was extracted using Trizol RNA isolation reagent (Invitrogen, Carlsbad, CA, USA) following the manufacturer's instructions. Before synthesizing the cDNA, the isolated RNA were added DNase I (Takara, Japan) and incubated for 30 min at 27°C for digesting DNA impurities. DNase I was then inactivated by heat treatment for 2 min at 80°C [5]. Then 4 µg of RNA were reversely transcribed to cDNA according to the manufacturer's instructions (Takara, Japan). Then the cDNAs were used as templates in PCR reactions with gene-specific primers (designed and synthesized by Sangon Biotech, Shanghai; Table S1). *UBQ* was used as a control in these reaction as described in the previous study [42]. The amplification reactions were performed in total volume of 20 µl. The Os *rboh* reaction was initiated at 94°C for 5 min followed by 30 cycles at 94°C for 15 s, 64.5°C for 1 min and 72°C for 1 min. The *UBQ* reaction was initiated at 94°C for 5 min followed by 30 cycles at 94°C for 15 s, 60.5°C for 1 min and 72°C for 1 min. The *PI3K* reaction was initiated at 94°C for 5 min followed by 30 cycles at 94°C for 15 s, 64.5°C for 1 min and 72°C for 1 min. The primers that were used in PCR were as follows: *PI3K* (F, AGGAGCCTAACTCGTGGAATAA; R, CACATACCAGCGAAGGAAGC), *UBQ* (F, CCAGGACAAGATGATCTGCC; R, AAGAAGCTGAAGCATCCAGC), Os *rboh*1 (F, AAGGGAATAACGGACGAAA; R, CCTCTGAACCACTCAAACG), Os *rboh*2 (F, CACAACTACCTAACAAGCGTC; R, TCCTCA CCTTGCTATCTCC), Os *rboh*3 (F, CTTTTGCTTATTGGTCTTGG; R, TTGTTTCGTGAGTGTAGGG), Os *rboh*4 (F, CAAGCGAGGTGTTTGTGGCA; R, GGCTGTCACCATACCACGGA), Os *rboh*5 (F, TTACTGCTGGTTGGATTAGGA; R, CATAGTAATGAGTGCTGACCGA), Os *rboh*6 (F, GAACGCTTGGCACGAAATAG; R, GAACGCTTGGCACGAAATAG), Os *rboh*7 (F, GTCAAATGCTTATGCTGTCA; R, TGTCCAGTCTCCGTTTGTT), Os *rboh*8 (F, CCCAGCAACCTCGGCTACAT; R, ACGCAGACGCAGTAGCCCAT), Os *rboh*9 (F, CCGTAAGGATTGAGAAGGT; R, GGGTCGTCGTAGATGTGGT).

### Determination of superoxide anion and ROS in rice seed embryos

ROS release from embryos was determined by the fluorescence of DCF, which was the product of oxidation of dichlorofluorescin diacetate (H_2_DCFDA), as described previously [43]–[45]. Briefly, the isolated embryos were cut longitudinally and incubated in 20 mM potassium phosphate buffer (pH 6.0) with H_2_DCFDA at a final concentration of 5 µM for 20 min at 30°C on a shaker. And then 200 µl of the solution was taken and its fluorescence (excitation: 488 nm, emission: 525 nm) was measured within a few minutes using a microtiter plate reader (Tecan, microplate reader, infinite M200; Austria) [46].

To determine superoxide anion, after imbibitions with or without some inhibitors, batches of 100 embryos were incubated in 1 ml of K-phosphate buffer (20 mM, pH 6.0) containing 500 µM XTT in darkness at 25°C on a shaker for 3 h, and XTT reduction was determined at 470 nm. Blanks without plant material were run in parallel and used for subtracting spontaneous fluorescence changes [43]. The accumulation of superoxide anion was also monitored in situ by NBT as described previously [6], [47]. Simply, seeds without capsules imbibed with DPI (100 µM) or LY294002 (60 µM) or Wortmannin (20 µM), and embryos were cut longitudinally and incubated with 6 mM NBT in 10 mM Tris-HCl buffer (pH 7.4) at 30°C for 2 h. Superoxide anion was visualized as deposits of dark-blue insoluble formazan compounds. Microscopic images were taken using a Zeiss microscope as described previously [48].

### Plasma membrane protein extraction from embryos

Plasma membranes were isolated by an aqueous two-phase partitioning system according to the method reported previously with minor modifications [8], [49]. The embryos were frozen in liquid nitrogen and fragmented using a pestle and mortar in a buffer (1.5 ml g^−1^ fresh weight) containing 0.25 M sorbitol, 1 mM EGTA, 20 µM phenylmethanesulfonyl fluoride, 1% (w/v) PVP, 2 mM dithiothreitol, and 50 mM Tris-acetate, pH 7.5, and filtered through four layers of gauze. The resulting filtrate was centrifuged at 15,000×*g* for 20 min. The recovered supernatant was centrifuged at 100,000×*g* for a further 30 min and the resulting membrane pellet (microsomal membranes) was gently resuspended in 5 mM potassium phosphate buffer (pH 7.8) including 330 mM Suc, 5 mM KCl, 1 mM DTT, 0.1 mM EDTA and protease inhibitors. The suspension was then fractionated by the aqueous two-phase partitioning method according to the batch procedure [50]. Plasma membrane vesicles were prepared using a 10 g aqueous two-phase partitioning system. Resuspended microsomal fractions were mixed with 6.5% (w/w) PEG 3350, 6.5% (w/w) Dextran T-500, 5 mM potassium phosphate buffer (pH 7.8) including 330 mM Suc, 3 mM KCl, 1 mM DTT, and 0.1 mM EDTA and protease inhibitors. After mixing, the phases were separated by centrifuging at 4,000×*g* for 5 min. The upper phase, enriched in plasma membrane (PM) vesicles, was repartitioned twice with fresh lower phase without PEG and, after dilution with 0.25 mM sorbitol, 1 mM EGTA, 2 mM MgCl_2_, 2 mM dithiothreitol, and 20 mM Tris-acetate, pH 7.5, and then the PM vesicles were collected by centrifuging at 100,000×*g* for 45 min. The pellets were then resuspended in Tris–HCl dilution buffer and stored at −80°C for further analysis. All procedures were carried out at 4°C.

### Determination of PM NADPH oxidase activity

The NOX activity of PM vesicles was determined by an assay based on reduction XTT by superoxide anion [34]. The analysis reaction medium contained 10 µg upper phase proteins, 0.3 mM XTT, and 0.18 mM NADPH in 1 ml 50 mM Tris-HCl buffer (pH 7.4) with 100 µM CaCl_2_ or 10 mM EGTA. Protein concentrations were determined by the approach of Bradford [51]. XTT reduction was determined at 470 nm in the presence and absence of 50 units SOD.

### In situ gel NBT assay

The NADPH-dependent superoxide anion coming from the isolated membrane fractions were assayed in native gels by a modified NBT reduction method [34], [52]. Protein samples from each fraction (20 µl per lane) were separated in a 7.5% (w/v) polyacrylamide separating gel and 4% (w/v) stacking gels with or without 0.1% (v/v) CHAPS at 4°C. The gel was incubated in the dark with 0.2 mM NBT solution (50 mM Tris–HCl, 0.2 mM NBT, 0.1 mM MgCl_2_, and 1 mM CaCl_2_, pH 7.4) for 20 min, and then with 0.5 mM NADPH until the appearance of blue formazan bands was observed. The reaction was stopped by immersion of the gel in distilled water.

### Western blot and coomassie staining of proteins

To obtain the optimal anti-body, amino acid sequences of Rac-1 in different species was compared using DNAman (Figure S3).

Membrane proteins and total proteins were separated by 12% SDS-PAGE and transferred onto PVDF membranes (American Pharmacia Biotech) and subjected to immunodetection with Rac-1/Cdc42 polyclonal antibody, which was the result of Rac-1 sequence comparison from different species. The Rac-1/Cdc42 antibody could recognize endogenous levels of total rac-1 and cdc42 protein. The antigen-antibody complex was visualized with anti-rabbit secondary antibody and enhanced chemiluminescence. The Coomassie Brilliant blue-stained gel was used to show that an equal amount of proteins [53]. All the experiments took three independent repetitions.

## Supporting Information

Figure S1 **H_2_O_2_ abate the inhibition of PI3K inhibitor on the rice seed germination.** Germination of rice seed embryos came from the same seed lot at 27°C in the dark. Rice seed treated with 10 mM KI, 1 mM ASA, 100 µM DPI, 20 µM Wortmannin, 60 µM LY294002, KI and H_2_O_2_ combined (10 mM KI+10 mM H_2_O_2_), ASA and H_2_O_2_ combined (1 mM ASA+10 mM H_2_O_2_), DPI and H_2_O_2_ combined (100 µM KI+10 mM H_2_O_2_), Wortmannin and H_2_O_2_ combined (20 µM Wortmannin+10 mM H_2_O_2_), LY294002 and H_2_O_2_ combined (60 µM LY294002+10 mM H_2_O_2_), respectively. The germination of rice seed after 5 days imbibition was counted. Data are means of three replicates ± SD. * indicates the values that are significantly different from control (P<0.05).
(TIF)

Figure S2 **Treatment with PI3K inhibitors decreases intracellular ROS level based on the treatment of DPI.** Rice seed embryos was pretreated with 100 µM DPI, DPI and Wortmannin combined (100 µM DPI+20 µM Wortmannin), DPI and LY294002 combined (100 µM DPI+60 µM LY294002), respectively. ROS was determinated by H_2_DCFDA. DCF fluorescence was measured using a microtiter plate reader as described in Materials and Methods. Data are means of three replicates ± SD.
(TIF)

Figure S3 **Comparison of amino acid sequences of Rac-1 in different species.** To obtain the optimal anti-body, amino acid was compared using DNAman, and the protein sequences used correspond to the following GenBank ID (from top to bottom): AAA36537, CAA40545, AAC49851, CAD42726, AAA96980, BAA84492.
(TIF)

Table S1 List of Primers used in semiquantitative reverse transcriptase -polymerase chain reaction (RT-PCR). *rboh* gene was searched from the rice genome database and appraised nine genes. Compared with *vps*34 gene in Arabidopsis, *pi3k* gene was found from rice genome database. *ubq* gene was used as a control as described in the previous study.
(DOC)
