# Supplementary figures and images for: Phosphatidylinositol 3-Kinase Plays a Vital Role in Regulation of Rice Seed Vigor via Altering NADPH Oxidase Activity
Source: PLoS One. 2012 Mar 20;7(3):e33817. doi: 10.1371/journal.pone.0033817 (PMC3309022; doi:10.1371/journal.pone.0033817)

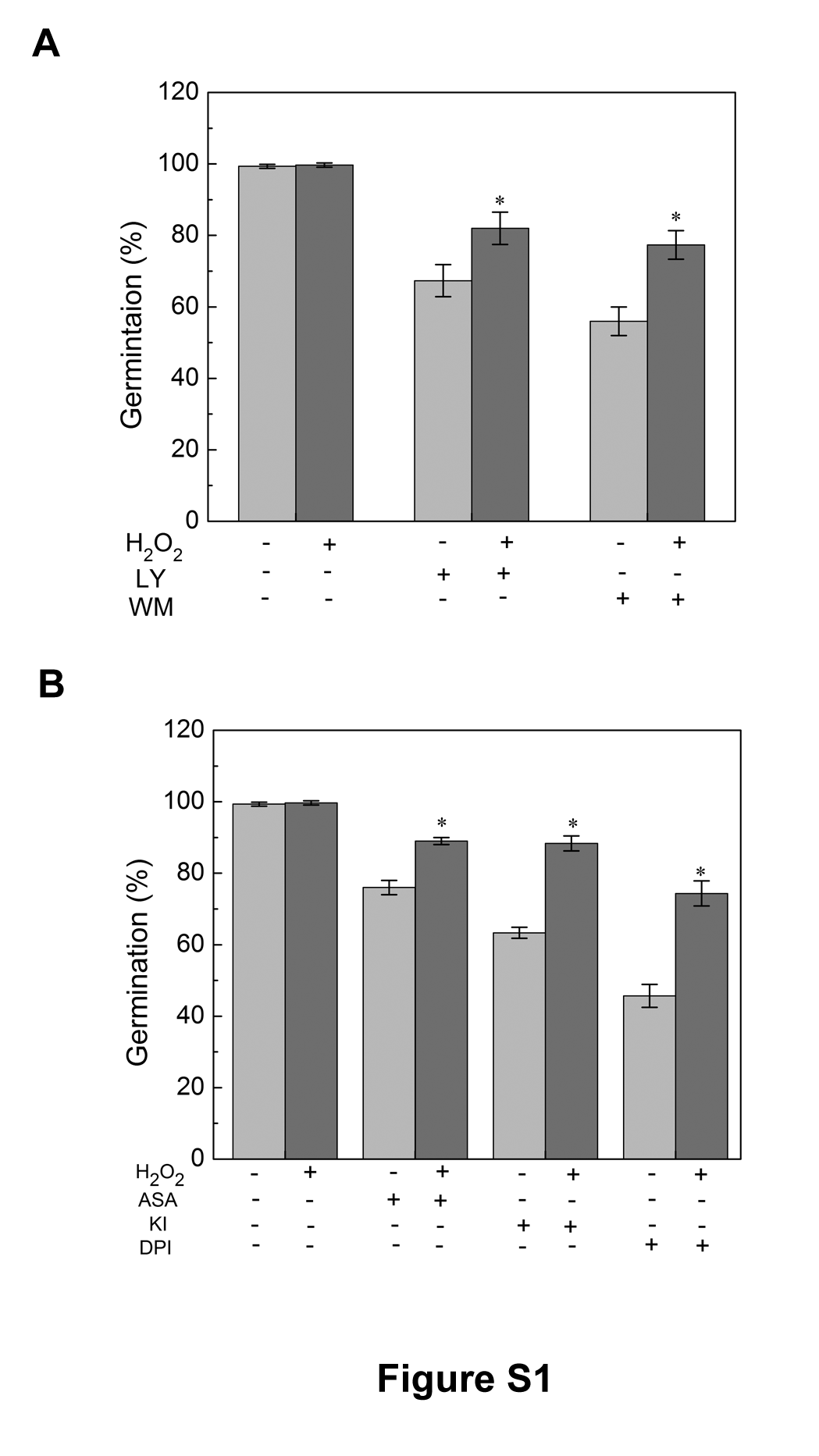

Supplement: Figure S1 — H2O2 abate the inhibition of PI3K inhibitor on the rice seed germination. Germination of rice seed embryos came from the same seed lot at 27°C in the dark. Rice seed treated with 10 mM KI, 1 mM ASA, 100 µM DPI, 20 µM Wortmannin, 60 µM LY294002, KI and H2O2 combined (10 mM KI+10 mM H2O2), ASA and H2O2 combined (1 mM ASA+10 mM H2O2), DPI and H2O2 combined (100 µM KI+10 mM H2O2), Wortmannin and H2O2 combined (20 µM Wortmannin+10 mM H2O2), LY294002 and H2O2 combined (60 µM LY294002+10 mM H2O2), respectively. The germination of rice seed after 5 days imbibition was counted. Data are means of three replicates ± SD. * indicates the values that are significantly different from control (P<0.05). (TIF) [file pone.0033817.s001.tif]

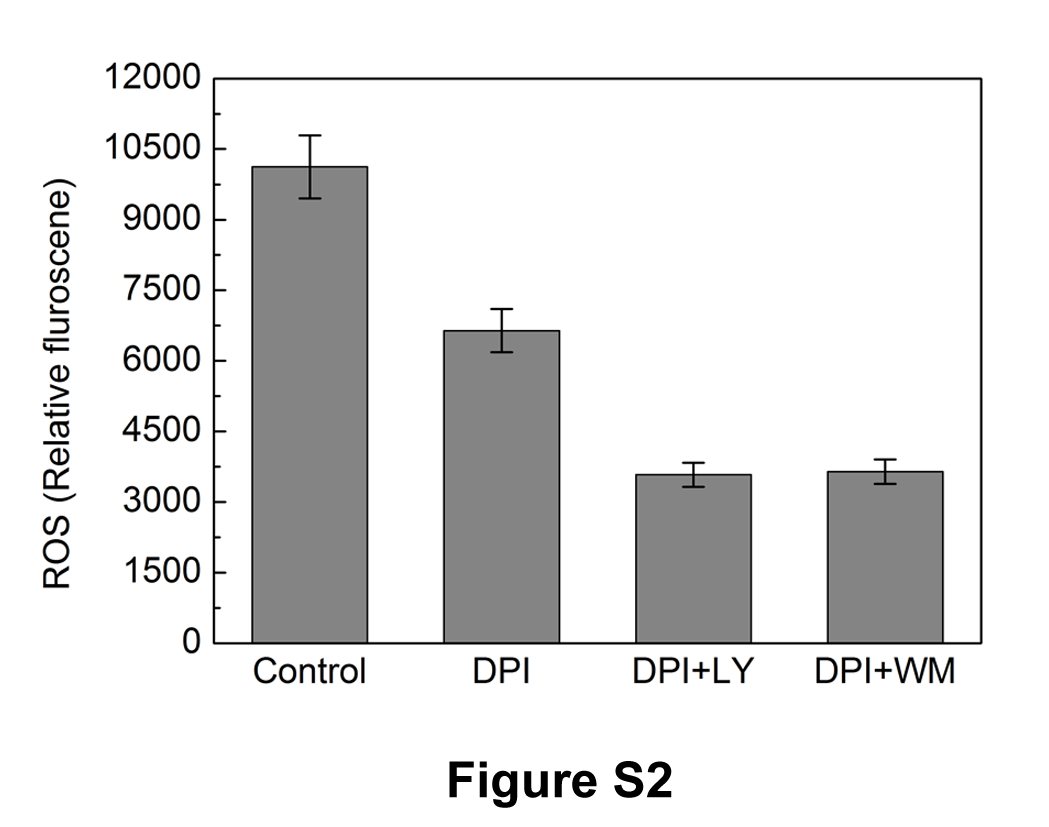

Supplement: Figure S2 — Treatment with PI3K inhibitors decreases intracellular ROS level based on the treatment of DPI. Rice seed embryos was pretreated with 100 µM DPI, DPI and Wortmannin combined (100 µM DPI+20 µM Wortmannin), DPI and LY294002 combined (100 µM DPI+60 µM LY294002), respectively. ROS was determinated by H2DCFDA. DCF fluorescence was measured using a microtiter plate reader as described in Materials and Methods. Data are means of three replicates ± SD. (TIF) [file pone.0033817.s002.tif]

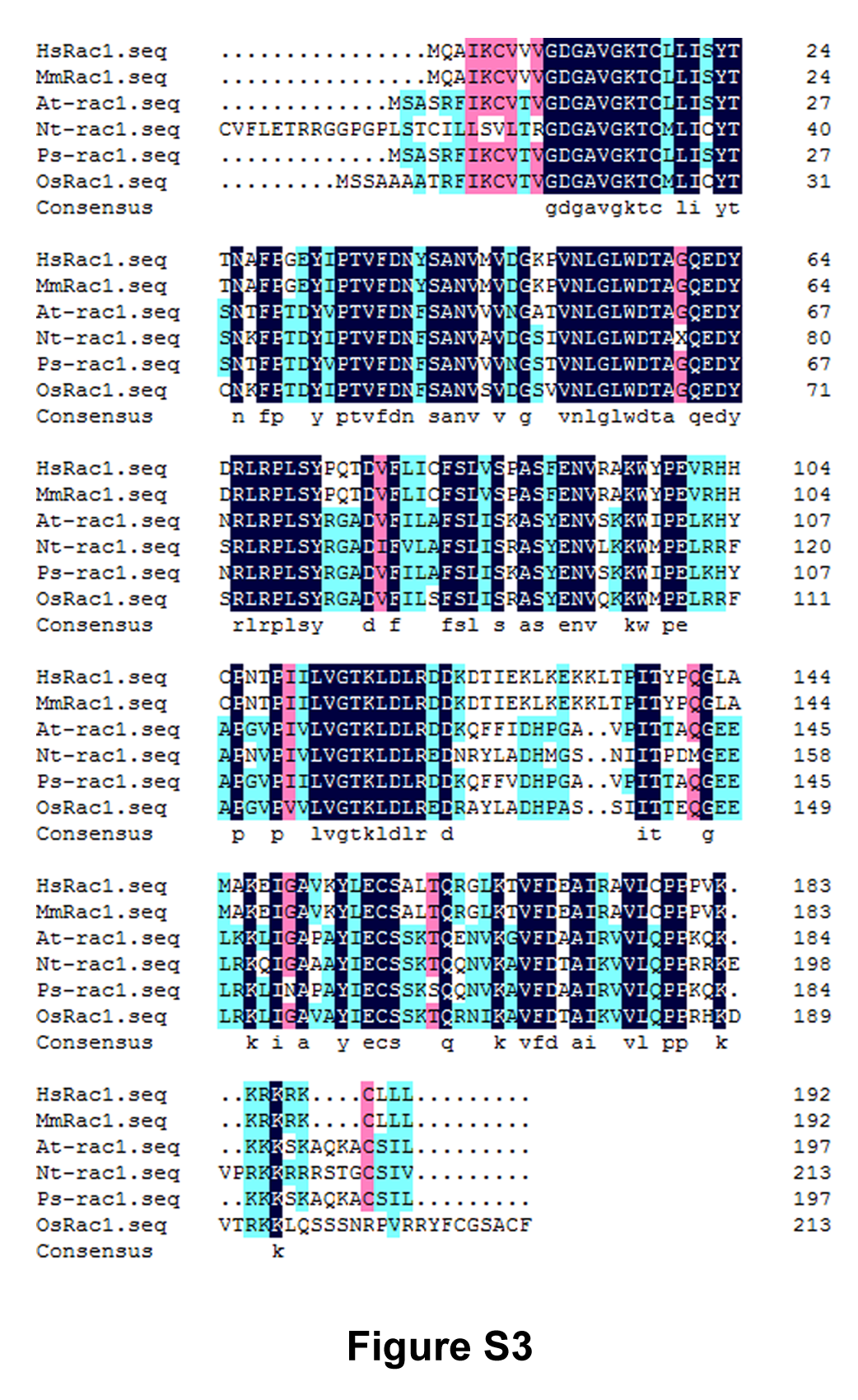

Supplement: Figure S3 — Comparison of amino acid sequences of Rac-1 in different species. To obtain the optimal anti-body, amino acid was compared using DNAman, and the protein sequences used correspond to the following GenBank ID (from top to bottom): AAA36537, CAA40545, AAC49851, CAD42726, AAA96980, BAA84492. (TIF) [file pone.0033817.s003.tif]
