# Supplementary material for: Phosphatidylinositol 3-Kinase Plays a Vital Role in Regulation of Rice Seed Vigor via Altering NADPH Oxidase Activity
Source: PLoS One. 2012 Mar 20;7(3):e33817. doi: 10.1371/journal.pone.0033817 (PMC3309022; doi:10.1371/journal.pone.0033817)
Supplement: Table S1 — List of Primers used in semiquantitative reverse transcriptase -polymerase chain reaction (RT-PCR). rboh gene was searched from the rice genome database and appraised nine genes. Compared with vps34 gene in Arabidopsis, pi3k gene was found from rice genome database. ubq gene was used as a control as described in the previous study. (DOC) [file pone.0033817.s004.doc]

**Table S1. List of Primers used in semiquantitative reverse transcriptase -polymerase chain reaction (RT-PCR).**

| **Gene** | **Accession no.** | **Forward primer (5’ to 3’)** | **Reverse primer (5’ to 3’)** | **Size (bp)** |
| --- | --- | --- | --- | --- |
| Os *rboh*1 | Os01g0360200 | AAGGGAATAACGGACGAAA | CCTCTGAACCACTCAAACG | 296 |
| Os *rboh*2 | Os01g0734200 | CACAACTACCTAACAAGCGTC | TCCTCACCTTGCTATCTCC | 411 |
| Os *rboh*3 | Os01g0835500 | CTTTTGCTTATTGGTCTTGG | TTGTTTCGTGAGTGTAGGG | 477 |
| Os *rboh*4 | Os05g0465800 | CAAGCGAGGTGTTTGTGGCA | GGCTGTCACCATACCACGGA | 344 |
| Os *rboh*5 | Os05g0528000 | TTACTGCTGGTTGGATTAGGA | CATAGTAATGAGTGCTGACCGA | 366 |
| Os *rboh*6 | Os08g0453700 | GAACGCTTGGCACGAAATAG | GAACGCTTGGCACGAAATAG | 360 |
| Os *rboh*7 | Os09g0438000 | GTCAAATGCTTATGCTGTCA | TGTCCAGTCTCCGTTTGTT | 233 |
| Os *rboh*8 | Os11g0537400 | CCCAGCAACCTCGGCTACAT | ACGCAGACGCAGTAGCCCAT | 314 |
| Os *rboh*9 | Os12g0541300 | CCGTAAGGATTGAGAAGGT | GGGTCGTCGTAGATGTGGT | 440 |
| *ubq* | D12629 | CCAGGACAAGATGATCTGCC | AAGAAGCTGAAGCATCCAGC | 245 |
| *pi3k* | AK111613 | AGGAGCCTAACTCGTGGAATAA | CACATACCAGCGAAGGAAGC | 483 |

**Table S1. List of Primers used in semiquantitative reverse transcriptase -polymerase chain reaction (RT-PCR).** *rboh* gene was searched from the rice genome database and appraised nine genes. Compared with *vps*34 gene in Arabidopsis, *PI3K* gene was found from rice genome database. *UBQ* gene was used as a control as described in the previous study.
